# Influence of early childhood parental hostility and socioeconomic stress on children’s internalizing symptom trajectories from childhood to adolescence

**DOI:** 10.3389/fpsyt.2024.1325506

**Published:** 2024-04-17

**Authors:** Lue Williams, Veronica Oro, Courtney K. Blackwell, Chang Liu, Elizabeth B. Miller, Jody Ganiban, Jenae M. Neiderhiser, David S. DeGarmo, Daniel S. Shaw, Tong Chen, Misaki N. Natsuaki, Leslie D. Leve

**Affiliations:** ^1^ Prevention Science Institute, University of Oregon, Eugene, OR, United States; ^2^ Department of Medical Social Sciences, Northwestern University Feinberg School of Medicine, Chicago, IL, United States; ^3^ Department of Psychology, Washington State University, Pullman, WA, United States; ^4^ NYU Grossman School of Medicine, New York University, New York, NY, United States; ^5^ Department of Psychological & Brain Sciences, George Washington University, Washington, DC, United States; ^6^ Department of Psychology, The Pennsylvania State University, University Park, PA, United States; ^7^ Department of Psychology, University of Pittsburgh, Pittsburgh, PA, United States; ^8^ Department of Psychology, University of California, Riverside, Riverside, CA, United States

**Keywords:** internalizing symptoms, parental hostility, socioeconomic stress, growth mixture modeling, childhood, adolescence, bioecological systems theory

## Abstract

**Introduction:**

Children and adolescents with elevated internalizing symptoms are at increased risk for depression, anxiety, and other psychopathology later in life. The present study examined the predictive links between two bioecological factors in early childhood—parental hostility and socioeconomic stress—and children’s internalizing symptom class outcomes, while considering the effects of child sex assigned at birth on internalizing symptom development from childhood to adolescence.

**Materials and Methods:**

The study used a sample of 1,534 children to test the predictive effects of socioeconomic stress at ages 18 and 27 months; hostile parenting measured at child ages 4–5; and sex assigned at birth on children’s internalizing symptom latent class outcomes at child ages 7–9, 10–12, 13–15, and 16–19. Analyses also tested the mediating effect of parenting on the relationship between socioeconomic stress and children’s symptom classes. Other covariates included parent depressive symptoms at child ages 4–5 and child race and ethnicity.

**Results:**

Analyses identified three distinct heterogenous internalizing symptom classes characterized by relative symptom levels and progression: low (35%); moderate and increasing (41%); and higher and increasing (24%). As anticipated, higher levels of parental hostility in early childhood predicted membership in the higher and increasing symptom class, compared with the low symptom class (odds ratio (OR) = .61, 95% confidence interval (CI) [.48,.77]). Higher levels of early childhood socioeconomic stress were also associated with the likelihood of belonging to the higher-increasing symptom class compared to the low and moderate-increasing classes (OR = .46, 95% CI [.35,.60] and OR = .56, 95% CI [.44,.72], respectively). The total (*c* = .61) and direct (*c’* = .57) effects of socioeconomic stress on children’s symptom class membership in the mediation analysis were significant (*p* <.001).

**Discussion:**

Study findings suggest that intervening on modifiable bioecological stressors—including parenting behaviors and socioeconomic stressors—may provide important protective influences on children’s internalizing symptom trajectories.

## Introduction

Internalizing problems in childhood and adolescence may be early indicators of functional problems associated with affective disorders, such as depression and anxiety (1, 2). Characterized by inwardly directed distress and maladaptive behavioral responses, early life internalizing symptoms are influenced by various bioecological factors. This includes parent-child relationships and socioeconomic status (3, 4); child characteristics such as age and sex assigned at birth based on anatomical and/or biological characteristics (5, 6); and common comorbidities that can have bidirectional effects on children and their environments, such as externalizing symptoms (7). In the United States, depressive disorders directly affect more than 20 million people (8), have a lifetime prevalence of 20.6% (9), and are among the most frequently reported sequelae of other diseases and serious health conditions (10). Depression prevalence among youth ages 12–17 is markedly lower, at 11.3%, than prevalence among adults (11). However, long-term negative outcomes are more pronounced when depressive conditions onset in childhood and adolescence, and annual rates continue to increase consistently (12, 13). Similarly concerning, in 2010, 272 million cases of anxiety disorders were reported worldwide, an increase of 36% over the preceding two decades, and overall prevalence was 4.0%, with the sharpest rise occurring among children and adolescents between ages 10 and 19 (14). A more recent meta-analysis reported a 20.5% global prevalence of anxiety among youth under age 18, which is nearly doubled since prior to the COVID-19 pandemic (15).

Longitudinal studies have used growth mixture and other latent class growth models to identify internalizing symptom development patterns, risks, and vulnerability factors across youth development (16, 17). Extant research has identified a diversity of growth patterns, including increasing, decreasing, early elevation, late onset, and low-stable growth patterns (17–21). Growth trajectories also vary according to their temporal windows. Studies that focus on internalizing symptom trajectories from early to late childhood (18, 20), and early adolescence to mid-adolescence (17, 22), and on anxiety and depressive symptom growth during these developmental periods (23, 24), have identified diverse factors impacting symptom development, including maternal psychopathology (18); parenting (20, 23, 25–28); child sex-assignment; and peer relations (17, 22).

Fewer studies have examined early childhood predictors of internalizing symptom progression (23) or temporal windows that include the range of critical developmental periods and transitions from childhood through adolescence (29). Analytical approaches in the present study were designed to fill this gap by examining the effects of socioeconomic stress and parental hostility as early childhood predictors (ages 18 months–5 years), in addition to the effects of sex assigned at birth, on symptom development that spans middle childhood (ages 7–9 and 10–12), middle adolescence (ages 13–15), and later adolescence (ages 16–19) using a large, geographically and racially diverse sample that is made possible by the cohort–wide data from the Environmental influences on Child Health Outcomes program (30).

### Internalizing symptom development as a bioecological process

The present study employed the Bioecological Systems Theory as a theoretical foundation. It allowed for the consideration of the independent and interconnected roles of the early childhood predictors (i.e., parental hostility and socioeconomic stress) and other meaningful correlates of the study’s outcome, including child sex assigned at birth, externalizing symptoms, race and ethnicity, and parent depressive symptoms. The model conceptualizes children’s development within and across nested systemic levels (31, 32). This includes the *individual/child* level at the center of the model; family members, peers, and local communities (*microsystem*); formal services, institutions, and environments (*exosystem*), such as healthcare systems and workplaces (33, 34); cultural norms, values, and ideologies (*macrosystem*) that influence exosystemic institutions and structures; and the interrelated contributions from each of these levels throughout a child’s development and across the life span (*chronosystem;* 35, 36). With the exception of mesosystemic social networks, which were not examined in the present study, all other bioecological levels are represented by a primary variable or covariate. An overarching anti-racist conceptualization was also employed at all levels of the research development process. Race and ethnicity, though not primary variables, are included in the analyses and discussion given the incontrovertible association between socioeconomic factors and race/ethnicity.

#### Parental hostility, a microsystemic factor

Multiple dimensions of parenting have been linked to children’s emerging internalizing symptoms, including intrusive and unresponsive parenting in early childhood. Parental hostility, one of two early childhood primary predictors examined in the present study, is characterized by non-supportive and controlling parenting practices, displays of anger and disappointment in children, punitive discipline, and perceived parental detachment (16, 37). Parental hostility toward children has been identified as a behavioral driver of many negative child outcomes (16, 38). For example, internalizing symptoms in both children and adolescents (ages 9–18) are predicted by harsh parenting (4, 7, 39) and childhood self-regulation and prosocial development problems (ages 6–7) are associated with hostile parenting in early childhood (ages 2–3; 40). Therefore, this investigation focused specifically on parental hostility during early childhood.

#### Socioeconomic stress, an exosystemic factor with macrosystemic influences

Associations have been identified between socioeconomic stress and risk for internalizing problems (3). Thus socioeconomic stress served as our second primary predictor. Socioeconomic stressors, as a function of lower socioeconomic status (SES), reflect appreciable disadvantages associated with various factors, such as household income (41, 42) and parent educational attainment (43). A family’s income, an exosystemic factor, has clear direct links to children’s mental health in the way it impacts children’s environments, including safe and secure housing, food access and nutrition, educational materials, quality healthcare, and childcare access (44–48). Parent educational attainment is another exosystemic contributor to SES that may influence employment options and social mobility that impact household finances and resources (44, 48). Socioeconomic stressors also indirectly influence children’s mental health through the microsystem. According to extant research, parent socioeconomic stress may have an indirect influence on child mental health and behavior via parenting (49, 50) and family interactions that are affected by finances (51, 52). It may also have indirect associations via macrosystemic ideologies and values that can shape an individual’s experiences based on racial and ethnic identities (51). In the present study, we used socioeconomic stress in early childhood as a predictor of child internalizing problems, and also examined its indirect effect via hostile parenting.

#### Sex assigned at birth, a vulnerability factor

Children’s internalizing symptoms and sex assigned at birth are individual-/child-level factors of the bioecological model. Furthermore, sex assignment is conceptualized in the present study as a potential vulnerability factor, rather than a risk factor. Studies examining internalizing symptom development among youth have identified important differences in vulnerability to higher-risk symptom development profiles among females as compared with males. Internalizing symptom trajectories in adolescent females follow heterogenous patterns that differ from symptom trajectories in adolescent males. Earlier and higher symptom peaks (29, 53–55) and higher symptom trajectories (29, 55–57), attributable to caregiver attachment, pubertal development, and other biopsychosocial differences, have also been observed among females as compared with males.

#### Correlates of internalizing symptom development: externalizing symptoms, parent mental health, and racial and ethnic identity

Externalizing symptoms share etiologies with children’s internalizing symptoms (58) and are a frequently co-occurring child characteristic that is important to account for when examining youth internalizing symptoms. Cascading models of development indicate that early externalizing behaviors may predict internalizing symptoms in later childhood (59). This developmental pattern highlights the importance of measuring and controlling for early externalizing symptoms when examining internalizing trajectories.

Parent-specific factors also contribute to microsystemic parenting behaviors and parent-child interactions that impact children’s internalizing symptoms. For example, parent mental health (e.g., depressive symptoms) has profound influences on the emotion regulation and brain development that play a central role in adolescent-onset depression (60, 61).

Macrosystemic factors related to racial and ethnic identity play a significant role in shaping sociocultural environments of children and their caregivers (36, 62–65). Research findings additionally highlight pervasive, cumulative impacts of systemically perpetuated adversity that place people of color in the United States at an unduly high risk for depression (54) and other long-term sequelae of early life internalizing problems. The current study thus included child race and ethnicity as a covariate in the primary analyses, while acknowledging and remaining attentive to the limitations inherent in controlling for factors in ways that potentially mask systemic disparities. In addition, child externalizing symptoms and parent depressive symptoms are included as covariates.

### The present study

Building from prior literature, the present study sought to identify risk factors for trajectories of internalizing symptoms from middle childhood through adolescence. We used the person-centered approach of growth mixture modeling to identify between-person differences in developmental trajectories that allow for the estimation of internalizing symptom group membership. Study aims included the investigation of the unique impact of two early childhood factors (parental hostility and family socioeconomic stress) on the progression of internalizing symptoms across critical developmental stages. Analyses to advance understanding of early life influences on childhood and adolescent internalizing behaviors were conducted, aligning with three hypotheses:

(1) Consistent with prior studies (e.g., 17–21), we hypothesized that 3–5 distinct developmental trajectories in children’s internalizing symptoms would be identified.
(2) Parental hostility and socioeconomic stress measured during early childhood, and sex assigned at birth, were hypothesized to predict internalizing symptom class membership; specifically, higher levels of parental hostility and socioeconomic stress during early childhood and female sex assignment were hypothesized to each be uniquely associated with membership in classes characterized by higher internalizing symptoms.
(3) A mediational path indicating the indirect effect of socioeconomic stress on internalizing symptom class membership mediated by parental hostility was hypothesized, in addition to the direct effect specified in hypothesis 2.

## Materials and methods

### Study design and procedure

The present study used data from two cohorts of the Environmental influences on Child Health Outcomes (ECHO) Cohort (30): the Early Growth and Development Study (EGDS; 66) and Family Life Project (FLP; 67). We combined these unique cohort data in order to increase the sample size and the diversity of sample characteristics, and to provide sufficient statistical power for the current analyses. Given the sample diversity, we also included cohort as a covariate to account for potential cohort-specific effects.

EGDS is an adoption design of 561 children who were adopted at birth and live in an adoptive home with genetically unrelated parents. Biological and adoptive caregivers were initially recruited through adoption agencies and enrolled in the study between 2003 and 2009. Assessment of family participants is ongoing and took place in 9-month intervals when adoptees were under age 3, and in 1-year and 2-year intervals from ages 3 through 18. EGDS also includes non-adoptees, but as the non-adoptees entered the study later in their development, they are not included in the current analysis. Eligibility required that families enrolled following the birth of an EGDS adoptee who lived in the adoptive home and whose biological parents were not deceased and who also agreed to participate in the research study (66).

The Family Life Project (FLP) is an ongoing, longitudinal study involving 1,292 families living in rural communities in eastern North Carolina and central Pennsylvania. FLP is a statistically representative stratified sample of every family with a mother that gave birth to a baby within the 1-year period between September 2003 and September 2004 while living within one of six predominantly low-wealth communities targeted for the study. African American families were oversampled to align with FLP research goals to examine the effects of economic resources, rural residency, and family relationships on youth development and better understand the effect of rural poverty and its intersection with race (68, 69). FLP participant families were assessed at baseline when children were 2 months old, during 2.5-hour home visits that included interviews, questionnaires, and observation of children and caregivers. Families were excluded from participation if English was not the primary spoken language and if the target child was not in the custody of the birth family (69, 70).

### Participant and sample characteristics

The EGDS sample included adopted children and their adoptive mothers and fathers. Among caregivers, there were 41 same-sex parent families (66). When the child was born, the median adoptive family annual income exceeded $100K (71). EGDS participants sampled for the present study included original study adoptees (*n* = 561 infants). Among the original adoptees, over half were male and White, 57.2% and 54.5%, respectively (66). Other racial and ethnic identities for the adopted children included 17.8% multiracial, 13.2% Black, 13.4% Hispanic/Latinx, and 1.1% Other Race/Ethnicity, including Asian and American Indian (66, 72). Given the relevance of caregiver characteristics in socioeconomic variables, some caregiver demographics are noteworthy. Average age of adoptive parents at the adoptee’s birth was 37.4 years (*SD* = 5.6). At the start of the study, 98% of adoptive parents identified as married. A majority of adoptive parents had attained higher education; 2-year college degree (8.0%), 4-year college degree (40.2%), and graduate school (38.8%; 66). Among the 481 EGDS participants sampled for the present study (80 of the original 561 adoptees did not have any data on the outcome measure and were thus not included in the analytical sample), 55% identified as non-Hispanic White, 30% identified as Latinx/Other, and 14% were non-Hispanic Black; 43% identified as female sex assigned at birth. Although the effects of adoption are not a focus of this study, prior published work with this adoption sample shows no detrimental effects of adoption on child development relative to children reared with their birth parents (66).

Among the 1,292 families in the FLP sample, participant racial demographics were proportional to demographic characteristics of the recruitment counties. Child participants were roughly equally divided between females (49%) and males. Among maternal caregivers, African Americans, primarily from North Carolina, accounted for 43% of the FLP sample and White caregivers represented the remaining 57%. Approximately 78% of families were considered low-income or economically poor based on reporting household income below two times the federal poverty line (73). Average caregiver age was 26 years (*SD* = 5.9) at the start of the study. Caregiver relationship status included married (48%), single (46%), and either separated, divorced, or widowed (6%). Eighty-one percent of caregivers either graduated from high school or earned a GED; 14% had earned at least a 4-year college degree (69, 70). Among the 1,053 FLP participants sampled for the present study, 50% identified as non-Hispanic White, 41% identified as non-Hispanic Black, and 9% were Latinx/Other; 49% identified as female sex assigned at birth.

### Measures

#### Internalizing symptoms

Internalizing symptom scale scores were measured at four time points–child ages 7–9 (*M* = 7.21, *SD* = 0.43); 10–12 (*M* = 11.17, *SD* = 0.59); 13–15 (*M* = 13.54, *SD* = 0.76); and 16–19 (*M* = 16.60, *SD* = 0.67). The Child Behavior Checklist for Ages 6-18 (CBCL/6-18; 74) instrument was administered to EGDS caregivers; the Strengths and Difficulties Questionnaire (SDQ-4 and SDQ-11; 75) was administered to FLP caregivers. We harmonized the CBCL/6-18 internalizing scale raw sum scores with internalizing scale raw sum scores from the SDQ-4 (for ages 4–10) and SDQ-11 (for ages 11–17) using validated cross-walk conversion tables developed with item-response theory (IRT) based equipercentile score linking procedures described in detail in a prior publication (see 76 for harmonization details). This enabled the SDQ scores to be expressed on the CBCL/6-18 metric to pool data for analyses.

The 119-item CBCL/6-18 assesses children’s internalizing and externalizing behaviors and social functioning between ages 6–18. Ninety-six and a half percent of adoptive caregiver reporters identified as female and mothers. Caregivers rated how well each item described their child within the past six months on a 3-point scale, including 0 (not true), 1 (sometimes true), and 2 (very true or often true). Raw sum scores for empirically derived syndrome scales were used to assess observed child internalizing characteristics and behaviors for the outcome measure. A 32-item empirically based internalizing syndrome scale comprised three subscales, including Anxious/Depressed, Withdrawn/Depressed, and Somatic Complaints. Parent reporters rated their children on attributes, behaviors, and affective symptoms that included items such as, “Feels worthless or inferior,” and “Withdrawn, doesn’t get involved with others.” Inter-item alphas have been found to be acceptable between caregiver raters. Cronbach’s alphas for the internalizing symptoms scale in the current study indicated good internal consistency reliability, α = .80. Prior validation study results also indicated acceptable test–retest reliability of repeated informant reports (77).

The Strengths and Difficulties Questionnaire for ages 4–10 (SDQ-4) and ages 11–17 (SDQ-11) utilize the same 0–2 scale as CBCL/6-18 items. The 25-item brief behavioral screening questionnaires were administered to parent reporters to assess child emotions and behaviors over the past six months. Ninety-five percent of caregiver reporters were birth parents who identified as female and mothers. Scores from the 5-item Emotional Problems scale and the 5-item Peer Problems scale were used to derive internalizing sum raw scores and, similar to the CBCL/6-18, assessed worries, mood, somatic symptoms, and social engagement as key facets of child and adolescent internalizing problems. Items included, “Many worries or often seems worried,” and “Often complains of headaches, stomach-aches or sickness.” The psychometric properties of the instrument are acceptable (75), including internal consistency reliability for different scores and reporters (mean Cronbach’s alpha = .73); interrater reliability of .86; higher interrater correlations between reporters above the meta-analytic mean used as a benchmark for both internalizing subscales (75); and high discriminant validity between community and psychiatric clinical samples (78).

#### Parental hostility

To measure parental hostility during early childhood (age 4–5; *M* = 4.16, *SD* = 0.28), we used items from the Parenting Scale (79). Specifically, the Parenting Scale was designed to identify parenting behaviors with known associations with child development and has been utilized in population studies examining associations between harsh discipline practices and children’s internalizing symptoms (80). Using a 30-item scale ranging from 1 (always) to 7 (never), caregivers reported the frequency of affectionate and combative interactions with their children over the past month. Items from a subset of the Overreactivity subscale were used, which included each item in the 3-item Hostility subscale (81, 82) and 5 additional items reflecting a wider range of hostile parent behaviors toward children related to criticism, shouting, and anger. Items include “When my child misbehaves, I spank, slap, grab, or hit my child,” “When I’m upset or under stress, I am picky and on my child’s back,” and “When my child misbehaves, I raise my voice or yell.” Item scores were summed to derive an augmented hostility scale ranging from 0 to 7 with higher scores indicating more parental hostility. Internal consistency reliability for the 8-item hostility subscale was minimally acceptable (α = .64).

#### Socioeconomic stress

Socioeconomic status-related variables of household income and educational attainment were measured in early childhood, between child ages 18 and 27 months (*M* = 21.11, *SD* = 2.07) and based on early childhood data available from both study cohorts. The income factor, measured at child ages 18 and 27 months and averaged, was the total combined household income (including wages, salaries, self-employment income, government assistance, interest, and dividends) of all household members that contributed to household expenses during the last calendar year. Five-category values for annual income ranged from 1 (less than $30,000) to 5 ($200,000 or more). Parent educational attainment, measured at child age 27 months, was a six-category variable that assessed the highest level of school completed, ranging from 1 (less than high school) to 6 (master’s, professional, and/or doctoral degrees). A socioeconomic stress score was computed across both cohorts as a reverse–scored standardized (z-score) mean composite of household income and the educational attainment of one parent reporter, where higher scores indicated greater socioeconomic stress. When available, income and education data from a second parent reporter were also included and a mean score of both parents was computed.

#### Child sex assigned at birth

Child sex assigned at birth was assessed using demographic data collected for both cohorts during the study enrollment period. Sex assigned at birth categories include female-assigned and male-assigned.

### Covariates

#### Child externalizing symptoms

Child externalizing symptoms were assessed at child ages 7–9 using the same measurement approaches to assessing internalizing symptoms in the present study and the same instrumentation, which included the CBCL/6-18 and SDQ-4 (i.e., harmonized externalizing score from parent reports). A 35-item externalizing syndrome scale from the CBCL/6-18 was drawn from two subscales, the Rule-Breaking Behavior and Aggressive Behavior subscales. Items include, “Breaks rules at home, school, or elsewhere” and “Temper tantrums or hot temper.” Harmonized externalizing scale items from SDQ-4 Conduct Problems and Hyperactivity/Inattention subscales include, “Often loses temper” and “Easily distracted, concentration wanders.” As aforementioned, psychometric properties of both the CBCL/6-18 and SDQ instruments are acceptable (75, 83).

#### Parent depressive symptoms

Caregiver depressive symptoms were measured using the Center for Epidemiologic Studies Depression Scale (CES-D; 84), a widely used 20-item adult self-report that evaluates depressive symptoms. We derived a mean symptom score assessed at child ages 4–5 years. Caregivers self-rated the frequency of symptoms they experienced over the past week using a 4-point scale that ranges from 0 (rarely or none of the time) to 3 (most or all of the time). Items respondents rated included, “I felt depressed,” “I felt that everything I did was an effort,” “My sleep was restless,” and “I thought my life had been a failure.” Substantial evidence for construct validity was reported, and included reasonable discriminant validity with scales designed to assess depressive symptoms; excellent concurrent validity by clinical and self-report criteria; high internal consistency in non-clinical samples (α = .85); and test-retest stability was acceptable for most non-clinical populations (*ICC* = .40–.70), with the exception of African Americans and the under 25 age group (84). Other validation studies confirm the instrument’s validity and suggest that ethnic differences among African American adults may be relevant to some construct factors (85).

#### Child race and ethnicity

Child racial and ethnic identities were assessed through demographic data collected for both cohorts during the study enrollment period and aggregated into mutually exclusive categories including race and ethnicity; Black/African American, White, any participants that identified as Latina/o/e/x and/or Hispanic, and/or any other race or ethnicity.

### Statistical analysis

Missing data patterns were evaluated using the *naniar* package version 0.6.1 (86) in R version 4.1.0 (87). Among 1,534 participants, there were 30 missing data patterns for variables in the covariance matrix. There were 541 participants (35%) with complete internalizing symptom data at all time points and 993 with partial data (65%). Across all primary study variables, there were 513 participants with complete data (34%), 371 were missing one of seven variables (24%), and 650 were missing two or more (42%). Data were not missing at random [Little’s MCAR χ^2^ (262) = 1764.00, *p* <.001]. Therefore, growth mixture and multinomial logistic regression models used full information maximum likelihood (FIML) estimates with robust standard errors (MLR). Comparisons among participants with partial and complete data revealed that participants with partial data were more likely to identify as Black [χ^2^(1, N =1534) = 5.78, *p* = .02]. There were no significant differences in missing data patterns for internalizing symptom sum scores, parental hostility, child sex assigned at birth, or cohort membership.

**Figure 1:**
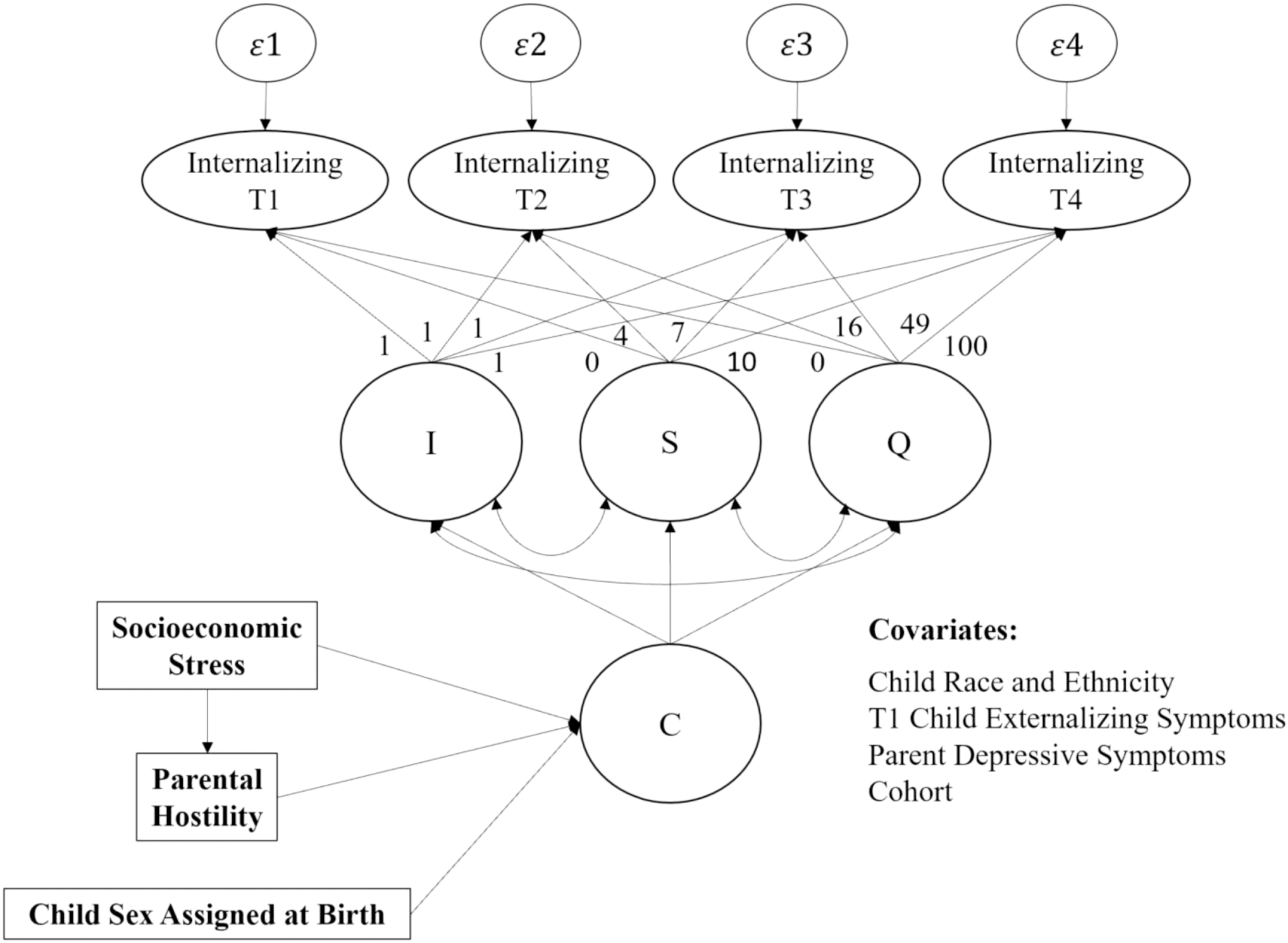
Latent Class Growth Mixture Model to Identify Developmental Trajectories of Internalizing Symptoms. I, intercept; S, slope, Q, quadradic slope; C, categorical variable for latent class. Conceptual model of all study variables used in analyses, including path coefficients for the latent class growth mixture model, intercept parameters fixed at 1, and slope coefficients fixed according to average time intervals (in years) between time 1 and time 2 (4 years), between time 1 and time 3 (7 years), and between time 1 and time 4 (10 years).

**Figure 2:**
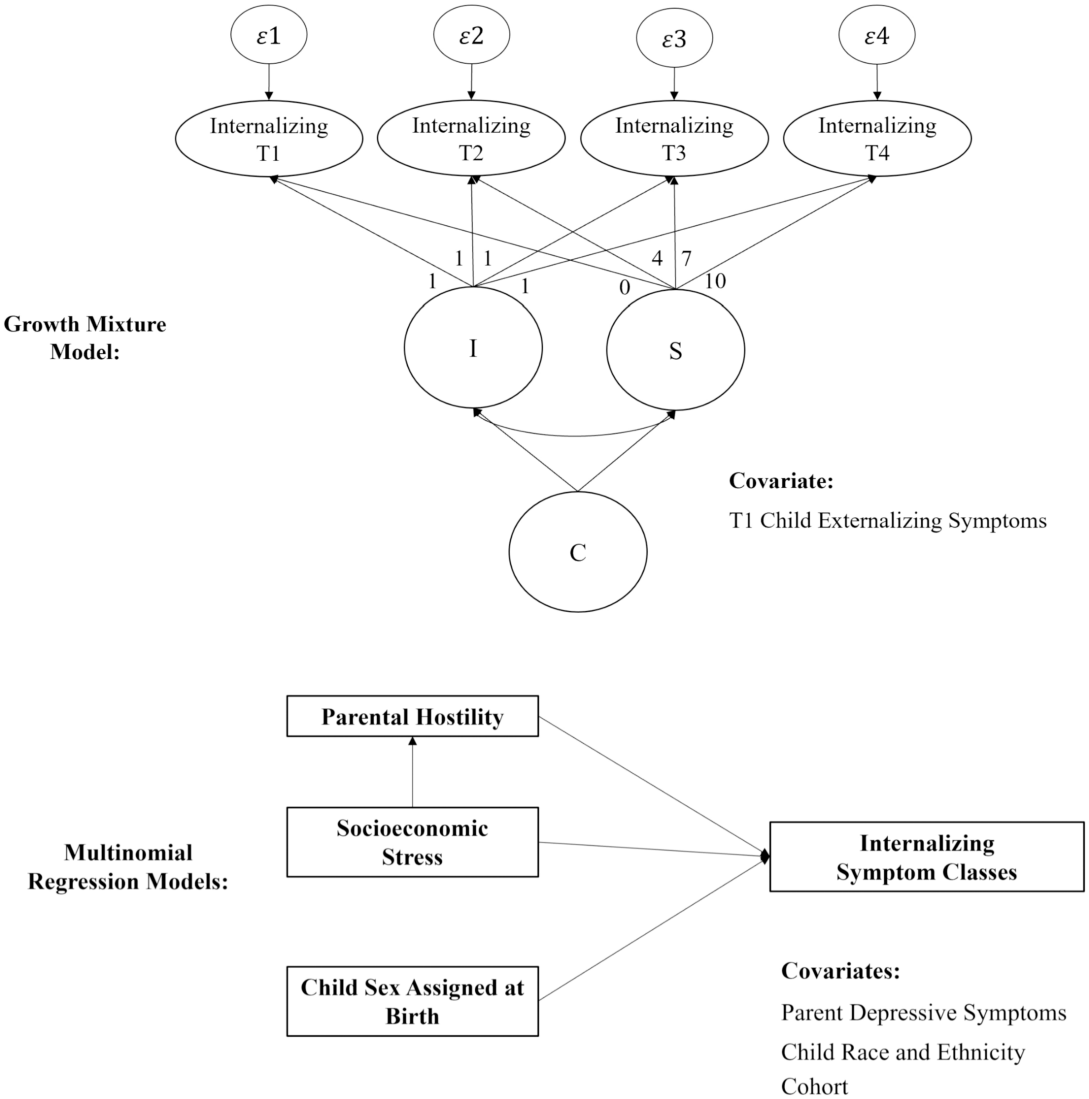
Simplified Path Diagrams of Growth Mixture Model and Multinomial Regression Models. I, intercept; S, slope, Q, quadradic slope; C, categorical variable for latent class. Growth mixture model diagram represents the latent class growth model used to estimate internalizing symptom classes. Multinomial regression models diagram represents analyses used to regress one categorical predictor, sex assigned at birth, and two continuous predictors, parental hostility, and socioeconomic stress, onto the outcome variable, internalizing symptom latent classes. The mediational regression analysis testing the indirect effect of socioeconomic stress on the outcome, is represented by the upward vertical arrow between the two measured continuous predictor variables, socioeconomic stress, and parental hostility.

To test the first hypothesis, growth mixture modeling (**Figures 1**, **2**) was conducted in Mplus version 8.8 (88) to identify distinct developmental trajectories of internalizing symptoms across four time points spanning middle childhood (T1, ages 7–9; T2, ages 10–12), early adolescence (T3, ages 13–15), and late adolescence (T4, ages 16–19). Full information maximum likelihood (FIML) was used to account for missing data (89). The growth mixture model comprises a univariate latent growth curve of internalizing symptoms formed by observations at T1, T2, T3, and T4 with an intercept (I) and slope (S), and a categorical variable for class (C); quadratic parameters were not estimated in the final models, as factor means and variances were nonsignificant across all classes. Time intervals between measurement occasions were not equally spaced; as such, slope factor loadings of 0, 4, 7, and 10 were specified to reflect years since T1 based on mean age. The zero factor loading at T1 defines the intercept growth factor as an initial status for internalizing symptoms. Intercept factor loadings were fixed at 1. A series of both unconditional and conditional models were tested as part of a multi-step approach wherein no covariates were initially considered, and T1 externalizing symptoms were later included as a covariate to account for comorbid presentations, respectively. Growth mixture models are a person-centered approach that completely model between- and within-person covariance structures, in contrast to latent class growth models which do not, by constraining the variance of the growth factors to zero and treating variability around the estimated classes as within-person error that is additionally constrained to be equal across time. Implementing such covariance equality constraints attenuates the distinctiveness of the classes and permits only mean differences in the within-class trajectories (90).

In order to determine the number of classes, an unconditional growth mixture model that included two classes was initially specified. Using an iterative approach, one additional class at a time was added and model fit was compared to that of the previous model to determine the best solution. Multiple fit indices were used to assess the number of classes; specifically, Bayesian Information Criterion (BIC) was used to narrow down the number of classes initially and the Vuong–Lo–Mendell–Rubin (VLMR) test was then used to further narrow the remaining plausible models (91). Moreover, successful convergence, entropy, percent of total count assigned to classes, and posterior probabilities were considered to determine the best class solution. This data-driven approach allowed for the probabilistic categorization of individuals into latent classes; each individual received a probability of belonging to each class and was assigned to their most likely class. Conditional models were then conducted, and class solutions were compared to the unconditional models. Class assignments from the final conditional model were saved and used in subsequent multinominal logistic regression and mediation analyses.

To test hypothesis 2, multinomial logistic regressions were conducted in Mplus version 8.8 (88) to examine the relationship between socioeconomic stress (measured at child ages 18 and 27 months), parental hostility (measured at child ages 4–5 years), and child sex assigned at birth, and likelihood of internalizing symptom trajectory class assignment, respectively. FIML was used to account for missing data. Probabilities of class assignments based on the aforementioned predictors were compared, with one class serving as the reference category to which probability of assignment to the other classes was compared. Covariates in all multinomial regression models included cohort; Black/African American, and Latinx and/or Hispanic and/or Other racial and ethnic groups dummy codes, with White as the reference group; and sex assigned at birth (when not treated as a primary predictor variable). Parent depressive symptoms were included as an additional covariate in tests of the association between parental hostility and class assignment. Based on the variations in EGDS and FLP participant characteristics, cohort was included in analyses as a control variable to account for racial and socioeconomic differences between the two cohorts that comprise the sample.

To test hypothesis 3, mediation analyses were conducted in Mplus version 8.8 (88) to examine whether parental hostility mediated the relationship between socioeconomic stress and internalizing symptom class membership. FIML was used to account for missing data. Sex assigned at birth, cohort, Black/African American, and Latinx and/or Hispanic and/or Other racial and ethnic groups dummy codes, with White as the reference group, were included as covariates on all paths in the mediation model. Parent depressive symptoms were included as a covariate on the b path, from parental hostility to internalizing symptom class assignment.

## Results

On average, children exhibited internalizing symptom raw sum scores of 6.64 (*SD* = 7.33, range: 0–59.70), 6.94 (*SD* = 6.80, range: 0–37.40), 7.70 (*SD* = 9.22, range: 0–64.20), and 7.86 (*SD* = 9.14, range: 0–61.50) at T1–T4, respectively. Among all children in the sample, 8% met borderline criteria for clinically significant internalizing symptoms (i.e., raw scores between 9–14 depending on age and sex assignment) and 14% met clinically significant symptom thresholds (i.e., raw scores greater than 12, 14, or 15 depending on age and sex assignment) at T1; 14% met borderline criteria and 13% met clinical thresholds at T2; 8% met borderline criteria and 16% met clinical thresholds at T3; and, at T4, 6% of children met borderline criteria and 17% met clinically significant thresholds for internalizing symptoms. On average, children exhibited externalizing symptom sum scores at T1 of 10.88 (*SD* = 13.31, range: 0–69.80). The mean score for socioeconomic stress in early childhood (ages 18–27 months) was 1.24 (*SD =* 0.90, range: -0.31–3.05). On average, parents reported hostility scores of 2.34 (*SD* = 0.80, range: 1–6.13) and depressive symptom scores of 0.54 (*SD* = 0.49, range: 0–2.65) when children were approximately 4–5 years of age. Descriptive statistics, including means, standard deviations, minimums, maximums, skewness, and kurtosis for all study variables are presented in **Table 1**. Cohort mean differences for all study variables were examined. FLP parents reported greater socioeconomic stress (*t* (1300.90) = -46.08, *p* <.001), less parental hostility (*t* (636.55) = 2.55, *p* = .011), and greater depressive symptoms (*t* (253.03) = -7.37, *p* <.001) than EGDS parents. FLP parents also reported that their children exhibited greater externalizing (*t* (1257.20) = -8.30, *p* <.001) and internalizing behaviors (*t* (1276.60) = -8.59, *p* <.001) at T1 than EGDS parents did.

**Table 1:** Study variable descriptive statistics.

	FLP						EGDS						Full Sample					
	N	%					N	%					N	%				
Female	521	49%					207	43%					728	48%				
Black	428	41%					69	14%					497	32%				
Latinx/Other	95	9%					146	30%					241	16%				
White	530	50%					266	55%					796	52%				
Participants	1053	69%					481	31%					1534	100%				
	Mean	SD	Min	Max	Skew	Kurtosis	Mean	SD	Min	Max	Skew	Kurtosis	Mean	SD	Min	Max	Skew	Kurtosis
EC SS	1.75	0.67	-0.14	3.05	-0.45	-0.18	0.34	0.44	-0.31	1.98	1.16	1.24	1.24**	0.90	-0.31	3.05	0.02	-1.22
EC PH	2.31	0.86	1.00	6.13	0.57	0.33	2.43	0.57	1.13	4.50	0.53	0.60	2.34*	0.80	1.00	6.13	0.52	0.52
EC PD	0.57	0.50	0.00	2.65	1.29	1.38	0.33	0.31	0.00	1.75	2.23	6.47	0.54**	0.49	0.00	2.65	1.40	1.73
T1 EXT	12.50	15.34	0.00	69.80	1.81	2.45	7.48	6.24	0.00	31.00	1.05	0.70	10.88**	13.31	0.00	69.80	2.17	4.51
T1 INT	7.60	8.25	0.00	59.70	2.20	7.03	4.61	4.21	0.00	24.00	1.36	2.00	6.64**	7.33	0.00	59.70	2.44	9.17
T2 INT	6.78	7.25	0.00	37.40	1.95	4.84	7.05	6.53	0.00	35.00	1.41	2.00	6.94	6.80	0.00	37.40	1.64	3.27
T3 INT	7.69	9.40	0.00	64.20	2.34	7.19	7.78	7.78	0.00	38.00	1.76	3.53	7.70	9.22	0.00	64.20	2.30	7.02
T4 INT	7.83	9.47	0.00	61.50	2.20	6.00	8.01	7.54	0.00	35.00	1.48	2.23	7.86	9.14	0.00	61.50	2.13	5.81

* p <.05 and ** p <.001 group difference between cohorts. Sex-assigned at birth coded 0 = male, 1 = female; Black = dummy coded race/ethnicity where 1 = Black/African American, 0 = other; Latinx/Other = dummy coded race/ethnicity where 1 = Latinx, and/or Hispanic, and/or other race/ethnicity, 0 = else; White = dummy coded race/ethnicity where 1 = White, 0 = other; EC, early childhood; SS, socioeconomic stress; PH, parental hostility; PD, parent depressive symptoms; T1 EXT, time 1 externalizing symptom values; T1 INT, time 1 internalizing symptom values; T2 INT, time 2 internalizing symptom values; T3 INT, time 3 internalizing symptom values; T4 INT, time 4 internalizing symptom values.

As indicated by zero-order correlations presented in **Table 2**, parental hostility was significantly and positively correlated with parent depressive symptoms (*r* = .33, *p* <.001) and was not significantly correlated with socioeconomic stress. Children whose parents experienced higher socioeconomic stress in early childhood exhibited significantly higher internalizing symptoms at T1 (*r* = .25, *p* <.001) and children exposed to more parental hostility in early childhood exhibited significantly higher internalizing problems at T1 (*r* = .21, *p* <.001), T2 (*r* = .15, *p* = .003), T3 (*r* = .12, *p* = .002), and T4 (*r* = .16, *p* <.001). Point-biserial correlations indicated that females exhibited significantly higher internalizing symptoms than males at T1 (*r* = .07, *p* = .02), T3 (*r* = .09, *p* = .02), and T4 (*r* = .10, *p* = .01) and significantly lower externalizing symptoms at T1 (*r* = -.13, *p* <.001). T1 externalizing symptoms were positively and significantly correlated with internalizing symptoms at all time points (*r* = .28 –.51, *p* <.001).

**Table 2:** Study variable zero-order correlations.

	1.	2.	3.	4.	5.	6.	7.	8.	9.	10.	11.	12.
1. FLP	–											
2. Female	.06*	–										
3. Black	.26***	.01	–									
4. Lx/Oth	-.27***	.01	-.30***	–								
5. White	-.05	-.02	-.72***	-.45***	–							
6. SS	.75***	.05	.42***	-.18***	-.26***	–						
7. PH	-.06*	-.02	-.01	-.03	.03	.00	–					
8. PD	.17***	.02	.09**	-.01	-.08*	.29***	.33***	–				
9. T1 Ext	.18***	-.13***	.06*	-.07*	-.01	.25***	.19***	.29***	–			
10. T1 Int	.19***	.07*	.11***	-.09**	-.04	.25***	.21***	.34***	.51***	–		
11. T2 Int	-.02	.01	-.03	-.06	.08	.06	.15**	.34***	.59***	.40***	–	
12. T3 Int	.00	.09*	-.09*	-.06	.13***	.06	.12**	.29***	.48***	.35***	.67***	–
13. T4 Int	-.01	.10**	-.01	.01	.00	.07	.16***	.30***	.42***	.28***	.53***	.67***

* p <.05; ** p <.01, *** p <.001; Cohort coded 0 = Early Growth and Development Study, 1 = Family Life Project (FLP) (n = 1,534); Sex-assigned at birth coded 0 = male, 1 = female (n = 1,534); Black = dummy coded race/ethnicity where 1 = Black/African American, 0 = other (n = 1,534); Other = dummy coded race/ethnicity where 1 = Latinx, and/or Hispanic, and/or other race/ethnicity, 0 = else (n = 1,534); White = dummy coded race/ethnicity where 1 = White, 0 = other (n = 1,534); SS, socioeconomic stress (n = 1,340); PH, parental hostility (n = 1,054); PD, parent depressive symptoms (n = 933); T1 Ext, time 1 externalizing symptom sum score (n = 1,284); T1 Int, time 1 internalizing symptom sum score (n = 1,284); T2 Int, time 2 internalizing symptom sum score (n = 463); T3 Int, time 3 internalizing symptom sum score (n = 744); T4 Int, time 4 internalizing symptom sum score (n = 734).

Fit indices for the conditional growth mixture models estimating internalizing symptom latent classes are presented in **Table 3**. An unconditional growth mixture model was used to estimate 2–5 class solutions. BIC and sample-size-adjusted BIC (SABIC) decreased through the 5-class model, whereas VLMR values indicated preference for the 3- and 5-class solutions over the 4-class solution. Average latent class probabilities indicated preference for the 3-class solution over the 4- and 5-class solutions, whereas entropy values indicated support for the 4-class solution over the 3- and 5-class solutions. For the conditional growth mixture models, BIC and SABIC decreased through the 5-class model, whereas VLMR values indicated preference for the 3- and 5-class solutions over the 4-class solution. Average latent class probabilities and entropy values indicated support for the 3-class solution over the 4- and 5-class solutions. Taken together, a 3-class solution was retained and latent class assignments from the 3-class conditional growth mixture model were saved and used in subsequent analyses.

**Table 3:** Fit indices for two- to five-class growth mixture models.

Fit Indices	2 Classes	3 Classes	4 Classes	5 Classes
AIC	29143.525	28600.417	28394.092	28271.404
BIC	29287.587	28819.178	28687.552	28639.563
Adjusted BIC	29201.814	28688.931	28512.831	28420.367
Entropy	.77	.71	.70	.68
LMR *p*-value	<.001	<.001	.064	.016
ALRT *p*-value	<.001	<.001	.066	.016

AIC, Akaike information criterion; BIC, Bayesian information criterion; Adjusted BIC, sample size-adjusted Bayesian information criterion; LMR, Vuong–Lo–Mendell–Rubin test; ALRT, Adjusted Lo–Mendell–Rubin test.

**Figure 3:**
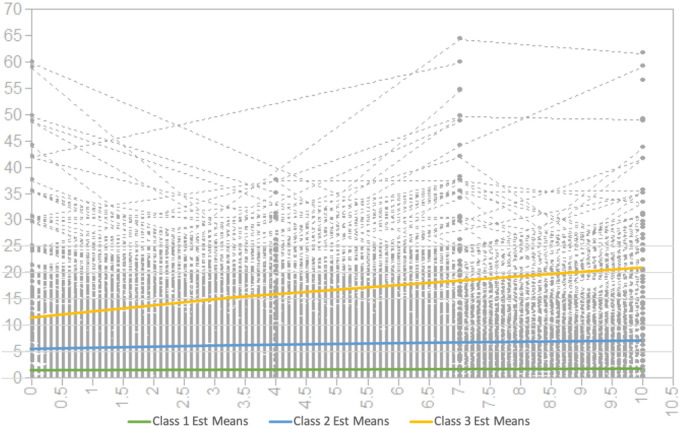
Three-Class Internalizing Symptom Trajectories. Gray lines represent 1,534 distinct observed growth trajectories of children’s internalizing symptom scores, as represented by the y-axis, with age represented (in years) by the x-axis between T1 (mean age = 7), T2 (mean age = 11), T3 (mean age = 14), and T4 (mean age = 17). The three prominent colored lines represent estimated mean internalizing symptom scores among children assigned to latent class 1, 2, and 3 based on heterogeneous symptom development.

**Figure 3** presents the observed individual values for internalizing symptoms and estimated internalizing means for classes 1–3. Mean scores for the growth factors of the 3-class conditional growth mixture model are presented in **Table 4**. The first class, comprising 35% of the sample, demonstrated the lowest level of internalizing symptoms with a significant mean intercept of 1.55 (*SE* = 0.20, *p* <.001) and linear slope of .03 (*SE* = 0.03, *p* = .38). The second class (41%) demonstrated a moderate level of internalizing symptoms with a significant mean intercept of 5.59 (*SE* = 0.53, *p* <.001) and significant slope of .16 (*SE* = 0.08, *p* = .04). Finally, the third class (24%) demonstrated a higher level of internalizing symptoms with a significant mean intercept of 11.51 (*SE* = 0.91, *p* <.001) and significant slope of .94 (*SE* = 0.18, *p* <.001). Class 1 exhibited a low and stable symptom trajectory, class 2 a moderate and slow increasing symptom trajectory, and class 3 a higher, accelerated growth symptom trajectory. Characteristics of the classes are presented in **Table 5**. Among children assigned to the lowest symptom class (class 1), none met borderline or clinically significant internalizing symptom thresholds at T1, T3, or T4; only one participant met borderline criteria at T2. Among children assigned to the moderate symptom class (class 2), 9% met borderline criteria for clinically significant internalizing symptoms and 6% met clinically significant symptom thresholds at T1; 20% met borderline criteria and 6% met clinical thresholds at T2; 9% met borderline criteria and 5% met clinical thresholds at T3; and at T4, 10% met borderline criteria and 5% met clinically significant thresholds for internalizing symptoms. Among children assigned to the highest symptom class (class 3), 16% met borderline criteria for clinically significant internalizing symptoms and 49% met clinically significant symptom thresholds at T1; 24% met borderline criteria and 48% met clinical thresholds at T2; 13% met borderline criteria and 47% met clinical thresholds at T3; and at T4, 8% met borderline criteria and 61% met clinically significant thresholds for internalizing symptoms. Participants assigned to class 3 had higher mean scores on early childhood socioeconomic stress and parental hostility, compared to classes 1 and 2. Females (compared to males) represented 46% of class 1, 49% of class 2, and 48% of class 3. Black children, comprising nearly one-third of the sample (32%), represented 28% of class 1, 32% of class 2, and 39% of class 3. Latinx/Other children (16% of the sample) represented 18%, 17%, and 10% of classes 1, 2 and 3, respectively. White children (52% of the sample) represented 54%, 51%, and 50% of classes 1, 2, and 3, respectively.

**Table 4:** Mean scores for growth factors of three-class growth mixture model.

Growth Factors	Class 1		Class 2		Class 3	
	Mean	*SE*	Mean	*SE*	Mean	*SE*
Intercept	1.55***	0.20	5.59***	0.53	11.51***	0.91
Linear Parameter	0.03	0.03	0.16*	0.01	0.94***	0.18

*** p <.001; * p <.05.

**Table 5:** Characteristics of internalizing symptom trajectory classes.

	Total Sample N = 1,534 (100%)	Class 1 N = 531 (35%)	Class 2 N = 634 (41%)	Class 3 N = 369 (24%)
	N (%)			
FLP	1053 (69%)	350 (66%)	407 (64%)	296 (80%)
Female	728 (47%)	242 (46%)	309 (49%)	177 (48%)
Black	497 (32%)	150 (28%)	202 (32%)	145 (39%)
Latinx/Other	241 (16%)	96 (18%)	107 (17%)	38 (10%)
White	796 (52%)	285 (54%)	325 (51%)	186 (50%)
T1 Bord. INT Symptoms	100 (8%)	0 (0%)	49 (9%)	51 (16%)
T1 Clin. Sig. INT Symptoms	186 (14%)	0 (0%)	31 (6%)	155 (49%)
T2 Bor. INT Symptoms	67 (14%)	1 (<1%)	42 (20%)	24 (24%)
T2 Clin. Sig. INT Symptoms	61 (13%)	0 (0%)	13 (6%)	48 (48%)
T3 Bord. INT. Symptoms	56 (8%)	0 (0%)	26 (9%)	30 (13%)
T3 Clin. Sig. INT Symptoms	120 (16%)	0 (0%)	15 (5%)	105 (47%)
T4 Bord. INT. Symptoms	45 (6%)	0 (0%)	30 (10%)	15 (8%)
T4 Clin. Sig. INT Symptoms	125 (17%)	0 (0%)	15 (5%)	110 (61%)
	Mean (SD)			
Socioeconomic Stress	1.24 (0.90)	1.08 (0.87)	1.17 (0.89)	1.60 (0.87)
Parental Hostility	2.34 (0.80)	2.12 (0.71)	2.35 (0.78)	2.57 (0.87)
Parent Depressive Symptoms	0.54 (0.49)	0.36 (0.35)	0.50 (0.44)	0.78 (0.58)
T1 Externalizing	10.88 (13.31)	2.90 (2.19)	7.90 (4.97)	26.69 (17.93)
T1 Internalizing	6.64 (7.33)	1.71 (1.48)	6.01 (3.81)	14.31 (9.93)
T2 Internalizing	6.94 (6.80)	2.42 (2.17)	6.50 (4.26)	14.76 (8.70)
T3 Internalizing	7.70 (9.22)	1.70 (1.58)	6.07 (4.41)	15.82 (12.22)
T4 Internalizing	7.86 (9.14)	1.91 (1.69)	6.57 (4.19)	18.47 (12.00)

Cohort coded 0 = Early Growth and Development Study (EGDS), 1 = Family Life Project (FLP); Sex-assigned at birth coded 0 = male, 1 = female; Black = dummy coded race/ethnicity where 1 = Black/African American, 0 = other; Other = dummy coded race/ethnicity where 1 = Latinx, and/or Hispanic, and/or other race/ethnicity, 0 = else; White = dummy coded race/ethnicity where 1 = White, 0 = other; Bord. INT Symptoms, Borderline Internalizing Symptoms; Clin. Sig. INT Symptoms, Clinically Significant Internalizing Symptoms; T1 Externalizing, time 1 externalizing symptom values; T1 Internalizing, time 1 internalizing symptom values; T2 Internalizing, time 2 internalizing symptom values; T3 Internalizing, time 3 internalizing symptom values; T4 Internalizing, time 4 internalizing symptom values.

A chi square test of independence was conducted to determine whether likelihood of class membership varied as a function of sex assigned at birth; results were nonsignificant (χ^2^ (2) = 1.21, *p* = .546). One-way ANOVAs were conducted to test for mean differences in socioeconomic strain, parental hostility, and T1-4 internalizing symptoms across classes. Mean levels of socioeconomic strain differed across classes (*F*(2, 1337) = 36.92, *p* <.001). A *post hoc* Tukey HSD test indicated that the mean for class 3 was significantly higher than both class 1 (95% CI, 0.37, 0.67) and class 2 means (95% CI, 0.29, 0.58); there was no significant difference between class 1 and 2 (95% CI, -0.22, 0.04). Mean levels of parental hostility differed across classes (*F*(2, 1051) = 25.48, *p* <.001). A *post hoc* Tukey HSD test indicated that the mean for class 3 was significantly higher than both class 1 (95% CI, 0.31, 0.61) and class 2 means (95% CI, 0.08, 0.36), and the mean for class 2 was significantly higher than the class 1 mean (95% CI, 0.10, 0.37). Mean levels of parental hostility differed across classes (*F*(2, 1051) = 25.48, *p* <.001). A *post hoc* Tukey HSD test indicated that the mean for class 3 was significantly higher than both class 1 (95% CI, 0.31, 0.61) and class 2 means (95% CI, 0.08, 0.36), and the mean for class 2 was significantly higher than the class 1 mean (95% CI, 0.10, 0.37). Mean levels of T1 internalizing symptoms differed across classes (*F*(2, 1281) = 467.09, *p* <.001). A *post hoc* Tukey HSD test indicated that the mean for class 3 was significantly higher than both class 1 (95% CI, 11.63, 13.57) and class 2 means (95% CI, 7.37, 9.22), and the mean for class 2 was significantly higher than the class 1 mean (95% CI, 3.46, 5.16). Mean levels of T2 internalizing symptoms differed across classes (*F*(2, 460) = 178.57, *p* <.001). A *post hoc* Tukey HSD test indicated that the mean for class 3 was significantly higher than both class 1 (95% CI, 10.79, 13.87) and class 2 means (95% CI, 6.80, 9.72), and the mean for class 2 was significantly higher than the class 1 mean (95% CI, 2.79, 5.35). Mean levels of T3 internalizing symptoms differed across classes (*F*(2, 741) = 220.46, *p* <.001). A *post hoc* Tukey HSD test indicated that the mean for class 3 was significantly higher than both class 1 (95% CI, 12.50, 15.75) and class 2 means (95% CI, 8.24, 11.27), and the mean for class 2 was significantly higher than the class 1 mean (95% CI, 2.85, 5.89). Finally, mean levels of T4 internalizing symptoms differed across classes (*F*(2, 731) = 342.90, *p* <.001). A *post hoc* Tukey HSD test indicated that the mean for class 3 was significantly higher than both class 1 (95% CI, 15.06, 18.07) and class 2 means (95% CI, 10.44, 13.36), and the mean for class 2 was significantly higher than the class 1 mean (95% CI, 3.35, 5.98).

Results from adjusted multinomial logistic regression models used to predict class membership by parental hostility, socioeconomic stress, and child sex assigned at birth to test hypothesis 2 are presented as odds ratios in **Table 6**. Multinomial regression models used to predict class membership by parental hostility indicated that the odds of being assigned to class 1 versus class 3 decreased by 39% for a one unit increase in parental hostility; parental hostility did not significantly predict class 2 versus class 3 membership. Significant covariate effects were such that odds of being assigned to classes 1 and 2 versus 3 decreased by 44% and 51% for FLP participants, respectively; and by 81% and 60% for a one unit increase in parent depressive symptoms, respectively.

**Table 6:** Odds ratios for predictors and likelihood of class membership.

	Class 1 vs Class 3	Class 2 vs Class 3
Variable	OR (95% CI)	OR (95% CI)
Female	0.94 (0.717, 1.228)	1.07 (0.83, 1.39)
Parental Hostility	0.61 (0.48, 0.77)	0.84 (0.69, 1.03)
Socioeconomic Stress	0.50 (0.35, 0.60)	0.56 (0.44, 0.72)

Sex assigned at birth was a covariate in all regression models where it was not treated as a predictor. Parent depressive symptoms was a covariate in the model testing the effects of parental hostility. Cohort and child race and ethnicity were covariates in all regression models.

Regression models that predicted class membership by socioeconomic stress indicated that the odds of being assigned to class 1 versus class 3 and to class 2 versus class 3 decreased by 54% and 44%, respectively, for a one unit increase in socioeconomic stress. Significant covariate effects were such that odds of being assigned to class 1 versus 3 increased by 67% for participants identifying as Latinx/Other compared to White participants.

Regression model results indicated that sex assigned at birth did not significantly predict class 1 versus class 3 membership or class 2 versus class 3 membership. Significant covariate effects were such that odds of being assigned to classes 1 and 2 versus 3 decreased by 45% and 52% for FLP participants, respectively.

**Figure 4:**
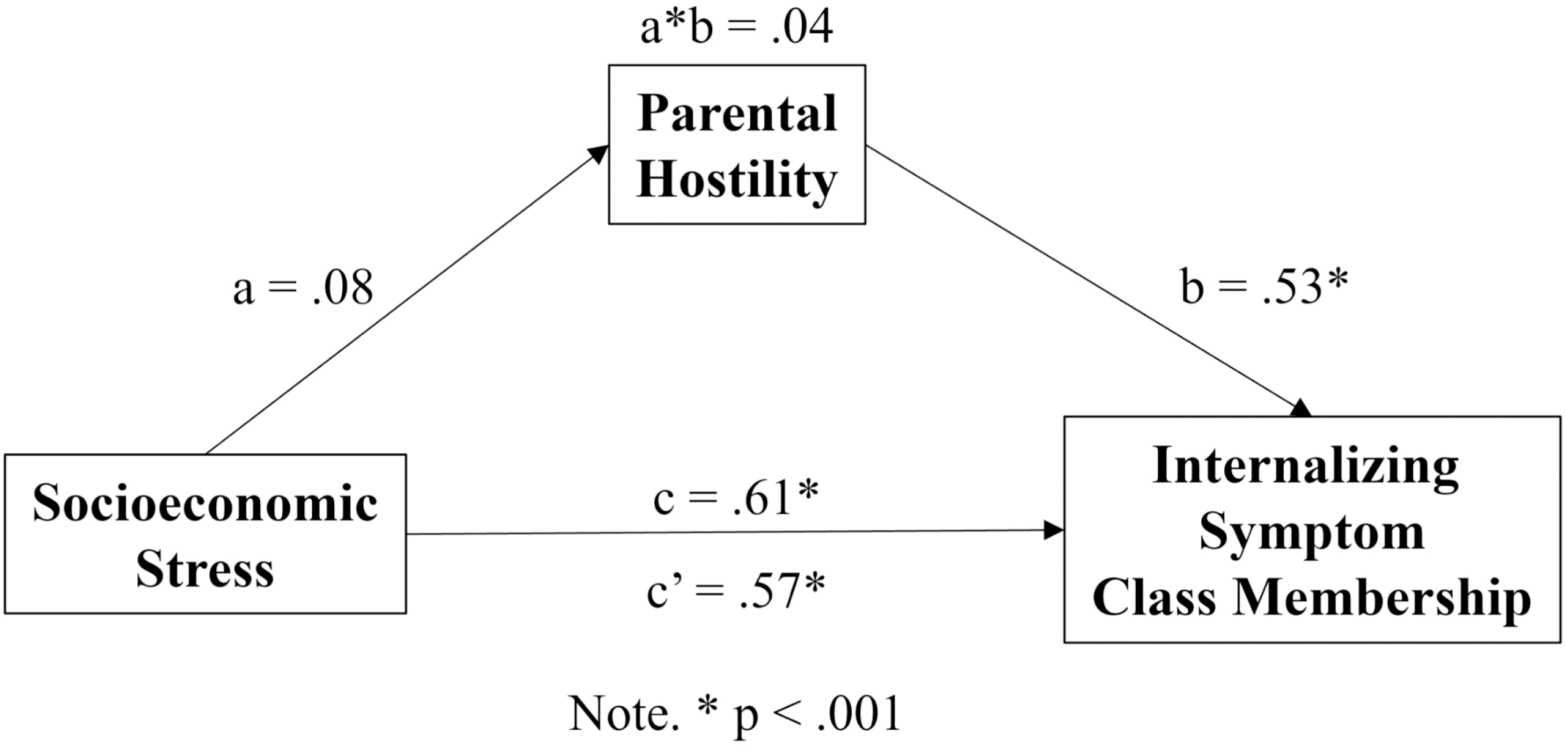
Mediation Model Contrasting Class 1 and Class 3 Membership. Mediational model depicting path coefficients where a represents the a path, b represents the b path, a*b represents the indirect effect, c represents the c path or total effect, and c’ represents the c’ path or direct effect of socioeconomic stress on the internalizing symptom class outcome variable.

The indirect effect of socioeconomic stress on class membership was tested for hypothesis 3 using mediation analysis contrasting class 1 and class 3 membership, as represented in **Figure 4**. The model indicated that the total effect of socioeconomic stress on class 3 membership was positive and significant (β = .61, *p* <.001). Socioeconomic stress was not significantly associated with parental hostility, although the association was at trend level (β = .08, *p* = .060). In turn, parental hostility was significantly associated with class 3 membership (β = .53, *p* <.001). The direct effect of socioeconomic stress on class 3 membership remained positive and significant, but the magnitude of the effect was attenuated (β = .57, *p* <.001). Finally, the indirect or mediated effect was not significant, though the estimate demonstrated a trend toward significance (β = .041, *p* = .082).

Mediation analysis contrasting class 2 and class 3 membership indicated that the total effect of socioeconomic stress on class 3 membership was positive and significant (β = 0.46, *p* <.001). Socioeconomic stress was positively and significantly associated with parental hostility in class 3 (β = 0.08, *p* = .042). In turn, parental hostility was not significantly associated with class 3 membership, although the association was at trend level (β = 0.20, *p* = .051). The direct effect of socioeconomic stress on class 3 membership remained positive and significant, but the magnitude of the effect was attenuated (β = 0.44, *p* = .001). Finally, the indirect or mediated effect was not significant (β = 0.02, *p* = .16).

Although models controlled for cohort, it is still possible that associations between our primary predictors and internalizing trajectories differed by cohort. Accordingly, we conducted supplementary analyses to test cohort interactions with parental hostility, socioeconomic stress, and sex assigned at birth. The only significant interaction was between cohort and socioeconomic stress, such that FLP participants under greater socioeconomic stress were more likely to belong to the higher and increasing internalizing symptom class than their EGDS counterparts. Mediation models indicated that cohort did not significantly moderate the path from socioeconomic stress to parental hostility nor did it in turn moderate the mediated or indirect effect. However, it did significantly moderate the total and direct effects of socioeconomic stress on internalizing symptom class membership, such that FLP participants under greater socioeconomic stress were more likely to belong to the higher and increasing internalizing symptom class than their EGDS counterparts. These results are provided in **Supplementary Tables 1** through **3**.

## Discussion

We identified three distinct heterogenous internalizing symptom classes (low-stable, moderate-increasing, and higher-increasing). Among the two higher symptom classes, significant symptom growth additionally characterized the developmental trajectories. These results demonstrate that symptom development patterns among children in the study sample are consistent with trajectories identified in extant research. Nearly two-thirds of children in the sample (65%) belonged to classes two and three, characterized by significant average increases in internalizing symptoms between middle childhood and late adolescence and moderate symptom development risk. The most pronounced risk was identified among participants in class 3, in which over 60% of children met clinical symptom thresholds by later adolescence. Results further indicated that risk is amplified among African American children, who were represented in a greater proportion in the highest symptom class (39%) as compared with their representative proportion among the full sample (32%). These findings align with the scientific literature, indicating the presence of disproportionate vulnerability and intersecting risk factors that may relate to racial identity, income, and rurality (67, 92). While these results underscore the importance of our decision to address racial differences in cohort composition, we also acknowledge key limitations to our approach, as it controlled for cohort differences in ways that could potentially mask the effects of racial disparities.

Notwithstanding these risks for children’s symptom development, study results also suggest that other factors may positively impact children’s symptoms. Over one-third of children in the sample (35%) belonged to the lowest symptom class with no detectable symptom growth, signaling potentially important factors of protection from the rearing environment. Parent depressive symptom and parental hostility scores among caregivers represented in the sample were low, according to psychometric cutoff scores of the validated instruments used, and may have contributed to an enriched rearing environment for some youth, providing important promotive effects.

### Influence of parental hostility and socioeconomic stress on class membership

Study results supported elements of hypothesis 2—hostile parenting behaviors predicted membership in the higher increasing symptom class. This indicates that harsh parenting behaviors are an important variable affecting children’s internalizing symptom trajectories. Parents who rely on hostility use guilt, humiliation, blaming, criticism, insults, coercion, and less reasoning in their approaches to discipline, which may perpetuate a child’s tendency toward internalizing symptoms (4, 93). Although average parent depressive symptoms were relatively low in the sample, their effect as a significant covariate negatively impacting children’s internalizing symptoms corresponds with extant literature linking parent psychopathology with child mental health outcomes (61).

Also consistent with hypothesis 2, the significant predictive effect of socioeconomic stress on the internalizing symptom class outcome indicates that there are exosystemic and macrosystemic factors that impact children’s psychological wellbeing. Variables used to measure socioeconomic stress in the present study indicate a meaningful influence of the socioeconomic environment by way of parents’ educational attainment and income that supports children’s households. Factors that influence the formal levels of education that parents may attain can have rippling impacts on employment options and vocational advancement (94, 95), as well as income.

Although the indirect effect of socioeconomic stress mediated by parental hostility (hypothesis 3) was not found to be statistically meaningful in the present study, analyses did not account for socioeconomic status and parent age as parent factors which could influence parental hostility. It is also possible that the constructs used to measure socioeconomic stress did not fully capture the array of bioecological stressors that could impact parenting behaviors in ways that exacerbate family stress and negatively influence child mental health (51, 96). Extant findings demonstrate that economic stress affects mental health outcomes via parenting behaviors among children and other members of a family system (50–52). However, the presence of other mediators, third variables, and complex influences from the macrosystem that confound the association are also plausible. While the present study did not demonstrate significant indirect influences of socioeconomic stress on child mental health, certain macrosystemic factors that impact children’s mental health indirectly through socioeconomic stress mediated by parenting can also have direct effects on socioeconomic stressors that affect households. Minority and acculturative stressors, for example, have well documented effects on myriad measures of health that disproportionally impact children and families of color with lower SES and other intersecting identities that confer risk (36).

Provided the present study’s guiding anti-racist framework, it is important to further acknowledge that systemic bioecological influences on caregivers’ experiences of their environments may have also contributed to potential cultural differences, some evidenced via cohort effects, on the relationships between primary predictors and child internalizing symptoms. Cultural and/or regional perspectives regarding discipline (97, 98) and the impact of identity-based acculturative stressors on parenting demands (51) may differentially impact families in rural environments, those experiencing economic hardship or poverty, and/or families exposed to racial and ethnic stressors. The significant protective effect of Latinx identity on the symptom class outcome further indicates that racial and cultural identity may have critical buffering effects on negative macrosystemic influences on the relationship between socioeconomic variables and children’s internalizing symptoms. Positive racial and ethnic identification (99), values and commitments to maintain family connections represented by familism (100), and perceived family resilience (101) have meaningful associations with positive child mental health outcomes, including internalizing and depressive symptoms, among Latinx children and adolescents. Although these effects were not tested in the present study, extant literature highlights the importance of attending to these factors in future research.

### Class membership predicted by child sex assigned at birth

Although we predicted, as part of hypothesis 2, that female sex assignment at birth would confer greater risk for internalizing symptom severity, the current study results deviated from expectations. Possible explanations for this result that were considered included the presence of positive features of the rearing environment (i.e., low levels of parent depressive symptoms and parental hostility) and parents’ gender-based expectations (102, 103) that could bias parents’ observation and interpretation of their children’s behaviors and symptoms. In addition, the parent-report nature of the internalizing symptom variable (rather than youth self-report, at the adolescent ages) may have played a role in this unexpected finding. We also consider, in study limitations, the possibility that the present study’s approach to measuring externalizing symptoms may not have fully accounted for differences by sex-assignment. However, assuming prior research studies examining the effects of sex-assignment on children’s internalizing symptoms may have shared similar protective sample characteristics and had similar exposure to gender-related sociocultural factors, it is likely that unknown features of the study sample—relevant in understanding the lower likelihood of membership in the higher symptom class among females—have not been fully explored in the present study.

### Study strengths and limitations

The present study harnessed a person-centered approach to examine the long-term developmental effects of certain risk and vulnerability factors associated with child and adolescent internalizing symptoms. Notwithstanding these strengths, limitations were also present. First, there are item-level differences between the early childhood and childhood versions of the harmonized CBCL and SDQ instruments. Thus, we were unable to use a measure of internalizing symptoms that included both early childhood and childhood/adolescent data. This meant that an early childhood internalizing construct—emotional reactivity—was not represented in our measure of internalizing symptoms for older children and adolescents.

A second study limitation was the use of parent reports to measure children’s internalizing symptoms. Although we chose to maintain consistency in the type of reporter used across outcome measurements, there is substantial evidence in the literature that child reports of their own symptoms, particularly during adolescence, are considered more reliable than parent reports (104, 105). With regard to the unexpected result that female sex assignment did not to confer higher risk for internalizing symptoms, it is plausible that children’s internalizing symptoms were more heavily influenced by commonly co-occurring externalizing behaviors or pre-existing externalizing symptoms that evoked harsher parenting responses prior to study outcome measurements (48).

In addition, limited availability of certain data across study cohorts resulted in systematic missingness and some instruments were not uniformly available and resulted in exclusion of certain constructs from statistical modeling. These limitations affected how constructs, such as socioeconomic stress, were operationalized. Income-to-needs (96, 106), for example, is a more sensitive assessment of family economic wellbeing than income alone; however these data were not available. Similarly, measures of economic strain (107) were not uniformly available in both sample datasets and could not be included in a composite variable measuring socioeconomic stress. This limitation was compounded by non-random missingness, where Black/African American participants were less likely to have complete data, and further highlights limitations related to how race and ethnicity variables were constructed and analyzed.

Similar to the construction of the socioeconomic stress variable, commonly used data aggregation approaches were employed to combine children’s racial and ethnic identity data used in analyses. However, race and ethnicity data aggregation approaches such as these, that group unique racial and ethnic communities into broader classifications of race and ethnicity, are aptly criticized for potentially masking important within-group differences (44, 108) and between-group disparities due to systemic racism (109).

### Future research directions

Future studies could employ various approaches to address study limitations.

First, to account for factorial invariance and item-level changes across repeated outcome measurements, second-order latent growth modeling and other shifting-indicator models, which were outside the scope of analyses for the present study, may be considered to ensure that the construct is being reliably measured across assessment time points (110, 111). Additionally, future studies that examine adolescent internalizing symptoms should utilize child-reported symptom data where feasible. To address the potential influence of externalizing symptoms on internalizing outcomes, future research could incorporate a measure of problematic child behavior prior to the measurement of hostile parenting.

With regard to data availability that limited access to useful variables, future investigations should consider including, if available, other important correlates of focal study constructs, such as pubertal development—given significant associations with internalizing symptom development (112–114) —and parental anxiety—associated with parenting behaviors and family conflict (115). Additionally, income-to-needs may be a preferred income-related factor contributing to the socioeconomic stress construct. Future studies may also consider other variables for their potential contributions to the construct, such as economic strain, and significant events associated with internalizing problems among adolescents, such as a parent’s job loss (106).

To address concerns related to aggregation of race and ethnicity data, alternative, nuanced approaches should be explored where possible. Analytical approaches that allow for comparisons between outcomes with and without controlling for racial and ethnic differences would shed important light on how discriminatory effects of structural racism may begin to impact children’s symptom trajectories in early life (109). Moreover, given the sociocultural influences of race, ethnicity, and other study constructs, future studies may also consider using theoretical frameworks that account for the diverse and intersecting effects of these macrosystemic factors. The Phenomenological Variant on Ecological Systems Theory (PVEST), for example, is a bioecological framework that additionally considers the influence of children’s phenomenological experiences as individual–level factors moderating effects from other levels of the bioecosystem (36, 116).

### Clinical and policy implications

Findings from the present study align with the scientific literature as to the role of parental hostility and socioeconomic stress on children’s internalizing symptoms and provide additional robust, longitudinal evidence that children’s psychological wellbeing is inherently related to the wellbeing of the people and communities with the most proximal bioecological influences on their development. Focusing exclusively on child-centered interventions may miss more sustainable, systems-based approaches to providing care for children’s caregivers and bolstering community supports (117). Findings from the present study emphasize the need for practitioners to engage with evidence-based interventions that effectively address and prevent children’s symptom development, integrate the needs of caregivers, and attend to structural barriers that impede access to child-centered, family-based, and preventative care. Policy reform efforts that engage anti-racist frameworks to address health outcomes more broadly further contribute by addressing system-wide and institutional barriers in ways that focus on the most vulnerable communities, families, and children (118). This means that accessible clinical approaches that target assessment and individual-level symptom reduction and management must operate in collaboration with the development and honing of effective preventative approaches to addressing social, environmental, and ideological contributors to long-term outcomes of childhood internalizing problems.

## Data availability statement

The datasets presented in this study can be found in online repositories. The names of the repository/repositories and accession number(s) can be found below: Eunice Kennedy Shriver National Institute of Child Health and Human Development (NICHD) Data and Specimen Hub (DASH): https://www.nih.gov/echo/cohort-data.

## Ethics statement

The studies involving humans were approved by Institutional Review Boards of the University of Oregon, the Pennsylvania State University, George Washington University, and the NYU Grossman School of Medicine. The studies were conducted in accordance with the local legislation and institutional requirements. The participants provided their written informed consent to participate in this study.

## Author contributions

LW: Conceptualization, Funding acquisition, Methodology, Project administration, Writing – original draft, Writing – review & editing. VO: Formal Analysis, Methodology, Writing – review & editing. CB: Methodology, Writing – review & editing. CL: Methodology, Writing – review & editing. EM: Writing – review & editing. JG: Funding acquisition, Investigation, Writing – review & editing. JN: Funding acquisition, Investigation, Writing – review & editing. DD: Methodology, Writing – review & editing. DS: Funding acquisition, Investigation, Writing – review & editing. TC: Writing – review & editing. MN: Funding acquisition, Investigation, Writing – review & editing. LL: Conceptualization, Funding acquisition, Investigation, Methodology, Writing – review & editing.
