# Supplementary material for: Influence of early childhood parental hostility and socioeconomic stress on children’s internalizing symptom trajectories from childhood to adolescence
Source: Front Psychiatry. 2024 Apr 17;15:1325506. doi: 10.3389/fpsyt.2024.1325506 (PMC11062022; doi:10.3389/fpsyt.2024.1325506)
Supplement: Supplementary file 1 [file DataSheet_1.pdf]

Supplementary Table 1

Odds Ratios for Predictor-Cohort Interactions and Likelihood of Class Membership

	Class 1 vs Class 3	Class 2 vs Class 3
Variable	OR (95% CI)	OR (95% CI)
Parental Hostility x Cohort	1.83 (0.60, 5.64)	1.51 (0.68, 3.35)
Parental Hostility	0.34 (0.11, 1.00)	0.57 (0.26, 1.22)
FLP	0.54 (0.37, 0.78)*	0.48 (0.34, 0.68)*
Female	0.95 (0.71, 1.27)	1.10 (0.84, 1.43)
Black	0.78 (0.56, 1.08)	0.94 (0.70, 1.26)
Latinx/Other	1.50 (0.94, 2.38)	1.41 (0.91, 2.19)
Parent Depressive Symptoms	0.19 (0.12, 0.30)*	0.40 (0.29, 0.56)*
Socioeconomic Stress x Cohort	0.36 (0.18, 0.70)*	0.54 (0.28, 1.03)
Socioeconomic Stress	1.05 (0.57, 1.92)	0.94 (0.52, 1.69)
FLP	0.82 (0.43, 1.57)	0.71 (0.38, 1.33)
Female	0.95 (0.72, 1.24)	1.08 (0.83, 1.40)
Black	1.12 (0.81, 1.56)	1.20 (0.88, 1.64)
Latinx/Other	1.78 (1.15, 2.76)*	1.57 (1.02, 2.41)*
Female x Cohort	1.70 (0.90, 3.19)	1.42 (0.77, 2.62)
Female	0.63 (0.36, 1.09)	0.81 (0.48, 1.37)
FLP	0.44 (0.28, 0.68)*	0.41 (0.26, 0.63)*
Black	0.74 (0.55, 1.00)	0.90 (0.68, 1.20)
Latinx/Other	1.43 (0.93, 2.20)	1.34 (0.88, 2.04)

Cohort coded 0 = Early Growth and Development Study (EGDS), 1 = Family Life Project (FLP); Sex-assigned at birth coded 0 = male, 1 = female; Black = dummy coded race/ethnicity where 1 = Black/African American, 0 = other; Other = dummy coded race/ethnicity where 1 = Latinx, and/or Hispanic, and/or other race/ethnicity, 0 = else.

Supplementary Table 2

Socioeconomic Stress Predicting Class 1 Versus 3 Membership, Mediated by Parental Hostility–Replication Including Socioeconomic Stress by Cohort Interaction

Outcome	β (*SE*)	*p*
Parental Hostility		
Socioeconomic Stress	0.19 (0.11)	.094
Cohort	-0.34 (0.12)	.004
Socioeconomic Stress x Cohort	-0.13 (0.12)	.302
Female	-0.02 (0.05)	.751
Black	-0.03 (0.06)	.578
Latinx/Other	-0.12 (0.08)	.109
Class 1 Versus 3		
Socioeconomic Stress	-0.47 (0.38)	.211
Cohort	0.49 (0.40)	.224
Socioeconomic Stress x Cohort	1.28 (0.42)	.002
Parental Hostility	0.55 (0.13)	<.001
Female	-0.01 (0.16)	.939
Black	-0.04 (0.19)	.840
Latinx/Other	-0.66 (0.26)	.010
Total effect		
FLP Socioeconomic Stress – Class 1 Versus 3	0.84 (0.17)	<.001
EGDS Socioeconomic Stress – Class 1 Versus 3	-0.37 (0.38)	.326
Indirect effect		
FLP Socioeconomic Stress – Parental Hostility – Class 1 Versus 3	0.04 (0.03)	.173
EGDS Socioeconomic Stress – Parental Hostility – Class 1 Versus 3	0.11 (0.07)	.118
Direct effect		
FLP Socioeconomic Stress – Class 1 Versus 3	0.80 (0.17)	<.001
EGDS Socioeconomic Stress – Class 1 Versus 3	-0.47 (0.38)	.211

Unstandardized coefficients are reported. Cohort coded 0 = Early Growth and Development Study (EGDS), 1 = Family Life Project (FLP); Sex-assigned at birth coded 0 = male, 1 = female; Black = dummy coded race/ethnicity where 1 = Black/African American, 0 = other; Other = dummy coded race/ethnicity where 1 = Latinx, and/or Hispanic, and/or other race/ethnicity, 0 = else.

Supplementary Table 3

Socioeconomic Stress Predicting Class 2 Versus 3 Membership, Mediated by Parental Hostility–Replication Including Socioeconomic Stress by Cohort Interaction

Outcome	β (*SE*)	*p*
Parental Hostility		
Socioeconomic Stress	0.20 (0.12)	.085
Cohort	-0.35 (0.12)	.004
Socioeconomic Stress x Cohort	-0.13 (0.12)	.294
Female	-0.02 (0.05)	.665
Black	-0.04 (0.06)	.540
Latinx/Other	-0.12 (0.08)	.103
Class 2 Versus 3		
Socioeconomic Stress	-0.19 (0.33)	.560
Cohort	0.45 (0.35)	.200
Socioeconomic Stress x Cohort	0.77 (0.37)	.034
Parental Hostility	0.21 (0.10)	.042
Female	-0.06 (0.14)	.665
Black	-0.24 (0.17)	.149
Latinx/Other	-0.50 (0.23)	.031
Total effect		
FLP Socioeconomic Stress – Class 1 Versus 3	0.60 (0.16)	<.001
EGDS Socioeconomic Stress – Class 1 Versus 3	-0.15 (0.33)	.646
Indirect effect		
FLP Socioeconomic Stress – Parental Hostility – Class 1 Versus 3	0.01 (0.01)	.226
EGDS Socioeconomic Stress – Parental Hostility – Class 1 Versus 3	0.04 (0.03)	.191
Direct effect		
FLP Socioeconomic Stress – Class 1 Versus 3	0.58 (0.16)	<.001
EGDS Socioeconomic Stress – Class 1 Versus 3	-0.19 (0.33)	.560

Unstandardized coefficients are reported. Cohort coded 0 = Early Growth and Development Study (EGDS), 1 = Family Life Project (FLP); Sex-assigned at birth coded 0 = male, 1 = female; Black = dummy coded race/ethnicity where 1 = Black/African American, 0 = other; Other = dummy coded race/ethnicity where 1 = Latinx, and/or Hispanic, and/or other race/ethnicity, 0 = else.
